# Approaching the nature of consciousness through a phenomenal analysis of early vision. What is the explanandum?

**DOI:** 10.3389/fpsyg.2024.1329259

**Published:** 2024-03-18

**Authors:** Bruno Forti

**Affiliations:** Department of Mental Health, Azienda ULSS 1 Dolomiti, Belluno, Italy

**Keywords:** explanatory gap, conscious structure, phenomenal analysis, explanandum, qualia, early vision

## Abstract

Loorits (2014) identifies the solution to the hard problem of consciousness in the possibility of fully analyzing seemingly non-structural aspects of consciousness in structural terms. However, research on consciousness conducted in recent decades has failed to bridge the explanatory gap between the brain and conscious mind. One reason why the explanatory gap cannot be filled, and consequently the problem remains hard, is that experience and neural structure are too different or “distant” to be directly compatible. Conversely, structural aspects of consciousness can be found in phenomenal experience. One possible alternative, therefore, is to seek the structure of seemingly non-structural aspects of consciousness not in the neural substrate, but within consciousness itself, through a phenomenal analysis of the qualitative aspects of experience, starting from its simplest forms. An essential premise is to reformulate the explanandum of consciousness, which is usually attributed to qualia and what it is like to be in a certain state. However, these properties do not allow us to identify the fundamental aspects of phenomenal experience. Sensations such as the redness of red or the painfulness of pain are inseparable from the context of the experience to which they belong, making qualia appear as phenomenal artifacts. Furthermore, the simplest qualitative aspects can be found in early vision. They are involved in perceptual organization and necessarily have relational significance. The unitary set of qualities found in early vision—such as those related to being an object, background or detail—constitutes the explanandum of the simplest forms of consciousness and seems to imply a justifying structure. Although early vision is characterized by interdependent qualitative components that form a unitary whole, we cannot find in it the structure of seemingly non-structural aspects of consciousness. Phenomenal appearance alone does not seem sufficient to identify a unitary structure of consciousness. However, the closeness of these characteristics to a unitary structure prompts us to delve into less explored territory, using the components of experience also as possible explanans.

## Introduction

In a 2014 paper, Loorits stated that “one possible way to present the hard problem of consciousness is to consider three seemingly plausible theses that are in an interesting tension. First, all the objects of physics and other natural sciences can be fully analyzed in terms of structure and relations, or simply, in structural terms. Second, consciousness is (or has) something over and above its structure and relations. Third, the existence and nature of consciousness can be explained in terms of natural sciences.” In other words, if we want consciousness to be explained in terms of natural sciences, we should be able to analyze it in structural terms. However, consciousness seems to be something that goes beyond its structure and relations. Loorits sees the possibility of analyzing in structural terms seemingly non-structural aspects of consciousness like qualia as the solution to the hard problem of consciousness.

Loorits founds his arguments on Crick and Koch’s work on consciousness (Crick and Koch, 1998). The idea is that the structure of a quale is a network of nodes (neurons) in the brain. A fully structural account of consciousness answers the question of how phenomenal consciousness could possibly “rise” from neural activity: if the hypothesis is correct, then the phenomenal consciousness simply is a certain complex pattern of neural activity. On this account a person experiences a particular quale when a given ensemble of neurons reaches a certain threshold.

However, this hypothesis does not seem capable of solving the hard problem, which is basically bridging the explanatory gap between physical properties and experience (Levine, 1983). It does not explain how a sensation could emerge from the activity of a network of neurons in the brain. Even in the way Loorits (2014) poses it, the hard problem seems to remain unresolved. In the years since Crick and Koch’s (1998) pioneering research, numerous authors have sought to identify the structure of Phenomenal Consciousness in neuronal organization (Seth and Bayne, 2022).

During the last three decades, the advent and development of new scientific procedures, such as functional magnetic resonance imaging and positron emission tomography, have allowed neuroscientists to study the activity of the living brain. These methods have been extensively used to identify with an acceptable degree of accuracy the neural correlates of any aspect of mental activity (Nani et al., 2019). Tracking the correlations between brain processes and states of phenomenal consciousness (neural correlates of consciousness) is the basic method of scientific consciousness research (Tononi and Koch, 2008; Polák and Marvan, 2018). Many potential neural correlates have been investigated. A classic example of an attempt to identity neural correlates of consciousness comes from the study by Sheinberg and Logothetis (1997), who used the phenomenon of binocular rivalry and significant correlation between neuronal activity and the conscious percept in infero-temporal cortex but not V1. My previous article (Forti, 2021) provides a detailed description of the correlations between brain processes and phenomenal consciousness. In short, one could say that consciousness is dependent on the brainstem and thalamus for arousal; that basic cognition is supported by recurrent electrical activity between the cortex and the thalamus at gamma band frequencies; and that some kind of working memory must, at least fleetingly, be present for awareness to occur (Calabrò et al., 2015).

With regard to subcortical structures, the cerebellum has four times more neurons than the cortex but has little effect on consciousness and its contents (Lemon and Edgley, 2010). By contrast, brainstem lesions typically cause immediate coma by damaging the reticular activating system and its associated neuromodulatory systems. However, neurological patients with a severely damaged cortex, but with relatively spared brainstem function, typically remain in a vegetative state. This suggests that brainstem activity is insufficient to sustain consciousness. Rather, it is likely that the activity of heterogeneous neuronal populations within the brainstem, hypothalamus and basal forebrain, which project diffusely to thalamic and cortical neurons and promote their depolarization, provides an important background condition for enabling consciousness by facilitating effective interactions among cortical areas (Parvizi and Damasio, 2001).

The role of the thalamus in consciousness remains controversial. Small bilateral lesions in the intralaminar nuclei of the thalamus can lead to coma, and chronic thalamic electrical stimulation may promote recovery in some patients with disorders of consciousness. Although the so-called core neurons in primary thalamic nuclei have focused connectivity, several higher-order thalamic nuclei are rich in widely projecting matrix cells, which may facilitate interactions among distant cortical areas. Thus, some thalamic cells may represent critical enabling factors for consciousness (Van der Werf et al., 2002; Koch et al., 2016).

With regard to cortical activity, according to the Global Neuronal Workspace model (Dehaene et al., 1998), when a stimulus is presented but not consciously perceived, activation can be seen mainly in the associated primary sensory cortices. When the stimulus is consciously perceived, however, activation in primary cortical areas is followed by a delayed ‘neural ignition’ in which a sustained wave of activity propagates across prefrontal and parietal association cortices (Noel et al., 2019) and send top-down signals back to all processors (Maillé and Lynn, 2020).

Other evidence across lesion, stimulation, and recording studies consistently point to regions in the “back” of the cortex, including temporal, parietal, and occipital areas, as a “posterior hot zone” that seems to play a direct role in specifying the contents of consciousness (Koch et al., 2016). By contrast, evidence for a direct, content-specific involvement of the “front” of the cortex, including most prefrontal regions, is missing or unclear (Boly et al., 2017). Although most prefrontal regions may be “mute” as regards to consciousness, it remains possible that some prefrontal regions, such as ventromedial areas (Koenigs et al., 2007) or premotor areas, may contribute specific conscious contents, such as feelings of reflection, valuation, and affect.

Recent neuroscientific findings challenge the widely held assumption that similar neural mechanisms underlie different types of conscious awareness, such as seeing, feeling, knowing, and willing. Even within a single modality such as conscious visual perception, the anatomical location, timing, and information flow of neural activity related to conscious awareness vary depending on both external and internal factors (He, 2023). For example, whether the prefrontal cortex is involved in conscious perception might depend on the characteristics of the sensory input: if it is simple and unambiguous, the prefrontal cortex might not be needed (DiCarlo et al., 2012); if it is complex or ambiguous, at least ventral prefrontal cortex appears to be recruited.

Some pathological conditions such as Contralateral Neglect syndrome could provide a window into consciousness. Jerath and Crawford (2014) suggest that the thalamus generates a dynamic default three-dimensional space by integrating processed information from corticothalamic feed-back loops, creating an infrastructure that may form the basis of our consciousness.

The impact of the circadian rhythms on spectral characteristics of EEG signals and on consciousness fluctuations has been investigated for more than half a century (Lehnertz et al., 2021). An activated or desynchronized EEG, one of the oldest electrophysiological indices of consciousness, is still one of the most sensitive and useful markers available (Koch et al., 2016). Spontaneous activity in the alpha-band may index, or even causally support, conscious perception (Gallotto et al., 2017). Low gamma-band (30–50 Hz) synchronization between neural groups coding the various features of objects currently populating experience has been proposed as a mechanism for dynamic functional integration in the brain and has been suggested to be the biological basis of perceptual experience and feature binding (Doesburg et al., 2009).

The time course of conscious perception has been studied using event-related potential components associated with awareness. Railo et al. (2011) argue that the visual awareness negativity component that occurs around 200 ms after stimulus presentation might be associated with conscious perception, and late positivity that occurs around 300–400 ms after stimulus presentation might be associated with conscious access (Raffone et al., 2014). Different event-related potentials likely correspond to different aspects of phenomenal consciousness—not all of consciousness—which may explain some of the disagreements in the literature (Friedman et al., 2023).

Recently, a number of theories have proliferated attempting to explain phenomenal experience and qualia based on the activity of electromagnetic field (Jones and Hunt, 2023). Field theories have arguably made real progress in explaining how fields integrate colors to form unified pictorial images (McFadden, 2020, 2023; Ward and Guevara, 2022). Theories of consciousness rooted in quantum physics are also well known (Hameroff and Penrose, 2014; Tuszynski, 2020). A major problem for quantum mind theories is to explain how quantum effects can occur in the brain at a sufficient scale to be useful (Tegmark, 2000; Bond, 2023).

However, all these studies do not seem to be able to bridge the explanatory gap between physical phenomena and phenomenal experience (Skokowski, 2022; Jones and Hunt, 2023; Sanfey, 2023). Neuroscientists track how light impinging on the retina is transformed into electrical pulses, relayed through the visual thalamus to reach the visual cortex, and finally culminates in activity within speech-related areas causing us to say “red.” But how such experience as the redness of red emerges from the processing of sensory information is utterly mysterious (Kanai and Tsuchiya, 2012). In other words, these studies do not seem capable of explaining the phenomenal and qualitative, seemingly non-structural aspects of consciousness. That is to say, they do not seem capable of bridging the explanatory gap between experience and physical substrate as is the case with the “qualitative” properties of wood and stone (Loorits, 2014).

In my opinion, a possible alternative is to look for the structure of seemingly non-structural aspects of consciousness not in the neuronal substrate, but in consciousness itself, through a phenomenal analysis of the qualitative aspects of experience, starting from its simplest forms. An essential prerequisite for this hypothesis is to define the explanandum in terms that can be useful for research. This article is aimed at defining the explanandum, i.e., what about consciousness we find useful to explain. In particular, I will try to highlight that qualia, which many authors identify as the main explanandum of consciousness, do not have a phenomenal existence as isolated entities and that the qualitative aspects analyzed in the literature must be placed in a more complex structural context than is commonly believed. Furthermore, the simplest qualitative aspects belong to early perception and necessarily have relational significance. This is a first step of a phenomenal analysis that I will develop further elsewhere, hypothesizing a hidden structure of consciousness.

## The problem of the specificity of consciousness

An often underestimated problem is the specificity of the aspects of consciousness that constitute the explanandum. In this sense, a theory of consciousness cannot avoid referring to qualia or, as I call them in this paper, the qualitative aspects of experience. The idea that consciousness has some features over and above its structural and relational properties has been strongly criticized by many (for example by most of the functionalists, behaviorists, and representationalists). However, most of the attempts to analyze consciousness in fully structural terms have ended up eliminating or simply ignoring certain (qualitative) aspects of consciousness whose existence is considered as absolutely obvious by many (Loorits, 2014). By eliminating or ignoring certain aspects of consciousness, these approaches to consciousness propose a correlation with something that is not necessarily conscious. In other words, one could say that they fail to identify the specificity of consciousness.

What aspects of consciousness that we recognize as such are useful in formulating a theory of consciousness? One way of asking this question is to ask what aspects of consciousness are specific, in order to avoid referring to “false positives,” i.e., states that are not conscious even though they exhibit some features typical of consciousness. The properties most often associated with consciousness (James, 1890; Tononi and Edelman, 1998; Zeman, 2001; Edelman, 2003; Searle, 2004) are the following: qualitative character; subjective; unitary; intentional; selective, with a foreground and background. According to Searle (2000), the essential trait of consciousness that we need to explain is unified qualitative subjectivity. Tononi and Koch (2015) identify five essential properties that belong to conscious experience, namely intrinsicality, composition, information, integration, and exclusion.

A fundamental distinction is the one between “Phenomenal” Consciousness and “Access” Consciousness (Block, 1995, 2005). Access consciousness can be considered a non-specific form of consciousness, as it can belong to consciousness, but also to many other non-conscious states (Tyler, 2020). Many theories of consciousness, as was historically the case with binding (Feldman, 2013), fail from square one precisely because they refer to something that is not specific to consciousness. Specificity is not fulfilled in the case of the unity of consciousness either, even though this is a characteristic that almost all authors attribute to consciousness. The unity of consciousness at a single time (Bayne, 2010), related to the ability to integrate information from all senses into one coherent whole—e.g. unified images (Jones and Hunt, 2023), can apply to different non-conscious systems. In Recurrent Processing Theory, the unconscious visual functions of feature extraction and categorizations are mediated by the feedforward sweep, while conscious functions related to perceptual organization are mediated by recurrent cortico-cortical connections (Lamme, 2010). However, these latter functions - that only occur when conscious percepts are present—are candidate neuronal correlates of consciousness. They are not conscious by themselves.

The Higher Order Theory of consciousness claims that a mere first-order representation is not sufficient for conscious experiences to arise (Brown et al., 2019). However, even a first-order state being in some ways monitored or meta-represented by a relevant higher-order representation is in no way sufficient for a state of consciousness to occur. The Global Neuronal Workspace model (Baars, 1997; Dehaene et al., 1998; Dehaene, 2014) is a model according to which conscious access occurs when incoming information is made globally available to multiple brain systems through a network of neurons with long-range axons. Why should global accessibility give rise to conscious experience (Chalmers, 2007)? Intentionality, as a quality of being directed toward an object, has often been associated with consciousness. But even a non-conscious system like an automaton can relate to something external to it. Not even the ability to select one region of the field as the object rather than another (Schwarzkopf and Rees, 2015) guarantees the occurrence of conscious experience. Therefore, there are aspects that do belong to consciousness, but not in a specific way. In the absence of specific features of consciousness, there is a risk of formulating a theory that refers to something that is compatible with the absence of consciousness.

Conversely, the specific characteristics of consciousness can be attributed to its phenomenal aspect. It is precisely this aspect of consciousness that is extremely difficult to explain in relational and structural terms. Phenomenal Consciousness (PC) seems to represent what is unique to consciousness, which exists exclusively in the presence of consciousness and not in other situations. Consequently, if a property such as unity undoubtedly applies to consciousness, then we should understand how the unity that manifests itself on the phenomenal level differs from other forms of unity (Wiese, 2017).

Difficulty arises when we try to better define the meaning of PC. How can we characterize phenomenal consciousness? Specificity is fulfilled if one experiences something in being an organism. According to Nagel (1974), a being is conscious just if there is “something that it is like” to be that creature, i.e., some subjective way the world seems or appears from the creature’s mental or experiential point of view. This is a vague and imprecise concept, presumably referring to a set of several closely intertwined components, such as more or less complex qualitative aspects, subjectivity and value connotations. As Loorits (2014) points out, “the most common ways to introduce the hard problem are intuitively appealing but rather obscure in meaning.”

A similar way of characterizing phenomenal consciousness is the notion of qualia (Kind, 2008). Qualia seem to fully meet the specificity criterion. The sheer qualitative feel of pain is a very different feature from the pattern of neuron firing that causes the pain (Searle, 1997). We shall see that the concept of quale, as interpreted by many authors, also appears questionable. In view of these limitations, in this paper I will refer to the concept of qualitative aspect rather than the concept of quale.

## Phenomenal analysis: investigating consciousness “from within”

The seemingly insurmountable difficulty of explaining the phenomenal aspects of consciousness must prompt us to reflect. We look for the structure of PC in the brain substrate, apparently without succeeding. However, we must ask ourselves whether the problem lies in consciousness itself rather than in the substrate. The extreme difficulty of explaining qualia in terms of brain structure could be considered an anomaly in the sense described by Lightman and Gingerich (1992). An anomalous fact is one that is unexpected and difficult to explain within an existing explanatory framework. According to Kuhn, awareness of anomaly is “the recognition that nature has somehow violated the pre-induced expectations that govern normal science.” In this sense, the structure of seemingly non-structural aspects of consciousness could be sought not in the neuronal substrate, but in consciousness itself. While it is known that consciousness has structural aspects, it is underestimated that many of them are related to its qualitative aspects. As I will try to highlight in this paper, the relational and unitary nature of its qualitative aspects cannot be ignored.

Experience and brain structure are too different or “distant” to be directly compatible. On the contrary, structural aspects of consciousness can be found in phenomenal experience. We can “perceive” the relational characteristics of PC. As will be discussed further below, despite the supposed intrinsic nature of qualia, many phenomenal aspects of experience—if not all—appear relational to us. At the same time, we can experience the unity of PC. The components of the perceptual field, such as the part and the whole, appear dependent on each other (Wagemans et al., 2012; Tononi and Koch, 2015).

Consequently, an analysis in structural terms of consciousness could be carried out not by searching for the structural features of the brain that can account for the phenomenal characteristics of consciousness (Tononi and Koch, 2015), but starting from the phenomenal properties of consciousness. There are phenomenal aspects that we do not usually take into account. It is important to point out that in almost all theories of consciousness, phenomenal aspects are either ignored altogether or are analyzed in a very cursory and superficial way.

The hypothesis of a structure of consciousness can only be explored by correctly identifying the starting point. This paper is devoted to the search for the explanandum—what about consciousness we find useful to explain, both in terms of specificity and simplicity. The explanandum of consciousness is usually traced back to qualia and what it is like to be in a certain state. However, the explanandum must be reformulated, since qualia, taken alone, are a phenomenal artifact. In addition, these properties do not make it possible to identify the basic aspects of phenomenal experience. Sensations such as the redness of red or the painfulness of pain must be placed in a more complex structural context than is commonly believed. The simplest qualitative aspects—such as those related to being an object, background or detail—can be found in early vision. Such phenomenal qualities, which are manifold and different from each other, are perceived in relation to each other and seem to form a unitary whole.

As I will explain later in the text, I am not referring to the most frequent definitions of early vision, which can start from retinal vision (Tomasi, 2006; Ghosh, 2020). Here I am referring to it as the simplest form of visual experience, related to perceptual organization. In this sense, early vision corresponds to Kanizsa’s (1979, 1980) “primary vision.” Early vision does not involve recognition, semantic interpretation, or other higher cognitive processing of visual information.

I call the method I adopt in this paper phenomenal analysis. Quite simply, its objective is to identify the structure of consciousness on the basis of the analysis of the phenomenal and qualitative aspects of experience, starting from its simplest forms. I call this analysis phenomenal rather than phenomenological because, while my approach has aspects in common with phenomenology in the observation of conscious phenomena, it does not aspire to embrace a methodological apparatus as complex as the one of phenomenology. My analysis primarily addresses very simple forms of experience, trying to prioritize the aspects that seem to belong to the fundamental “framework” of consciousness and might be involved in the formation of its structure.

Moreover, phenomenology investigates what characterizes perceptions, judgments or feelings. Its goals do not involve the search for an explanation of consciousness, as phenomenology addresses phenomena as they manifest themselves in the intentional consciousness of the subject. “Phenomenology is concerned with attaining an understanding and proper description of the experiential structure of our mental/embodied life; it does not attempt to develop a naturalistic explanation of consciousness, nor does it seek to uncover its biological genesis, neurological basis, psychological motivation, or the like” (Gallagher and Zahavi, 2008).

With respect to the matter of simplicity, it is worth noting that, in addition to identifying the specific aspects of consciousness, a theory of consciousness should identify the simplest forms of phenomenal consciousness. There are several reasons for this. First of all, in any theory it is important to identify the fundamental aspects of the phenomenon under study. The identification of elementary units has been a key in many fields of science and could also be a key in the field of consciousness research (Kanai and Tsuchiya, 2012). Secondly, it is necessary to identify the simplest level at which consciousness manifests itself. Edelman (2003) distinguishes between primary consciousness, which concerns sensations, images and perceptual experiences in general, and higher-order consciousness, which includes self-consciousness and language. However, the main problem is the description of primary consciousness, because higher-order consciousness emerges from processes that are already conscious. Thirdly, the simplest forms of consciousness might have been the first to appear in the course of evolution and the primary significance of its appearance should be traced to them. Finally, the most difficult aspects to explain seem to be the apparently less complex ones. In this sense, the mystery of consciousness seems to boil down to the impossibility of explaining the fact that we experience sensations (Chalmers, 1995). Simple aspects such as the redness of red or the painfulness of pain help identify the problem of consciousness very effectively (Humphrey, 2006).

I will focus phenomenal analysis not on qualia and raw feelings, but on the qualitative aspects of the simplest forms of visual experience taken as a whole. This way, phenomenal analysis makes it possible to highlight the relational nature of the qualitative aspects of perceptual experience. As we shall see in the course of the analysis, at some point there comes the problem of explaining how the qualitative components of the conscious field form a totality of interdependent parts. In fact, the different qualitative components of the phenomenal field appear to be both distinct and dependent on each other at the same time, without it being possible to identify which structure is responsible for this.

This appears to be a limitation of an analysis that considers only the apparent aspects of visual experience. However, the “closeness” of these characteristics to a unitary structure prompts us to delve into less explored territory, using the components of experience also as possible explanans. In a separate paper, starting from the nature of appearance itself, I will consider the need to postulate the existence of non-apparent aspects.

## Qualia are a phenomenal artifact

One of the main problems in the approach to consciousness is that we tend to identify the simplest aspects of experience with qualia. It is a common view that simple qualia could be a useful starting point for a theory of consciousness. Koch (2004) wonders how the elemental feelings and sensations making up conscious experience arise from the concerted actions of nerve cells and their associated synaptic and molecular processes. The assumption is that if we explain the neuronal substrate of pain, sweetness and the redness of red we lay the foundation for explaining consciousness.

However, identifying the simplest aspects of experience with qualia is erroneous. According to the majority of authors, considering qualia as a possible starting point for a theory of consciousness means being able to think of them as isolated, or extrapolating them from objects and other components of the field of experience as fully representative of experience itself. Then, it means being able to look for the simplest possible explanation of consciousness at the level of brain organization. It should be noted that, although Lewis separates the properties of qualia from those of objects, he does not identify them with conscious experience: “This given element in a single experience of an object is what will be meant by ‘a presentation.’ Such a presentation is, obviously, an event and historically unique. No identification of the event itself with the repeatable content of it is intended” (Lewis, 1929). However, the way in which literature on consciousness has defined the concept of quale over time has coincided with a tendency to separate it from anything having to do with the idea of relationship and structure. Qualia are intrinsic, i.e., non-relational (Dennett, 1988; de Leon, 2001; Siddharth and Menon, 2017). As Loorits (2014) points out, qualia are some features of consciousness over and above its structural and relational properties.

The meaning of non-relational is not univocal. We must distinguish between internal relations and external relations. Regarding the former, Dennett (1988) states that “qualia … are *intrinsic* properties—which seems to imply … that they are somehow atomic and unanalyzable.” Simple qualia such as blueness or sweetness have no obvious signs of an internal structure (Haun and Tononi, 2019). According to Loorits (2014), in the classical view, qualia would be monadic, not compositional, and with no internal structure: “when I have a visual perception of a red apple, I have a direct epistemic access to many structural features of my visual experience: the size and shape of the perceived apple, for instance. I do not have similar direct epistemic access to the structure of the perceived redness of my visual experience.”

However, it should be noted that the non-analyzability of qualia is related to the fact that they are characterized by an internal homogeneity, which Metzinger (2004) calls ultrasmoothness, in the sense that they have a grain structure. We should keep in mind that at the conscious level we can make a phenomenal distinction only by contrasting one region with another. If there is no contrast within a red surface, we perceive it as homogeneous and cannot make any phenomenal distinction. However, its supposed non-analyzability, which we perceive phenomenally as homogeneity, is a piece of information about the region of the perceptual field that differs from the possibility of any point or part of that region not being red. Experiencing the redness of red means seeing the red color distributed homogeneously over an object. This is information that we receive from experience and that we ignore if we speak abstractly about the redness of red. Therefore, the unanalyzability of qualia is at least questionable with regard to its internal relations.

With regard to external relations, according to de Leon (2001), “that qualia are intrinsic means that their qualitative character can be isolated from everything else going on in the brain (or elsewhere) and is not dependent on relations to other mental states.” According to the standard view, qualia are not in themselves, representational or intentional (Loar, 2002). According to Dennett (1988), intrinsic means that they are non-relational properties, which do not change depending on the experience’s relation to other things. Consequently, qualia would not be related:

1. with other mental states and behavioral output, so they are not mental states in the functional sense of the term (de Leon, 2001; Van Gulick, 2017);
2. with the stimulus and, in a broad sense, with the external reality to which they refer (see inverted or absent qualia), so they are non-intentional and non-representational (Loar, 2002);
3. with other components within the experiential field; or at least, they can be separated from them, e.g., from the object, so they are universals (Lewis, 1929; Dennett, 1988).

While the first two statements concern undeniably important aspects, specifically the functional and the intentional ones, the third is crucial for a phenomenal conception of consciousness. Claiming that qualia are not characterized by their relations to other components of the field has three implications: first, the idea that extrapolation from other components of the field can allow the phenomenal properties of qualia to be preserved; second, that everything within the field that has to do with relation is not, in the strict sense of the word, phenomenal; and third, that everything that has to do with relation, and more broadly with structure, can be explained in terms of cerebral or other organization.

However, relations with other components of the experiential field have to do with the very nature of experience, of what is phenomenal. In the absence of such relations, qualia risk being incompatible not only with a functional and intentional view of mind (Loar, 2002), but also with the essence of PC. Since qualia are extrapolated from the phenomenal experience in which they are placed, they give no guarantee of retaining phenomenal qualities, so they cannot be considered fully representative of the experience itself. The universality of qualia, i.e., the possibility of their being recognized from one experience to another, must be distinguished from their phenomenal nature, which is related to their relations in each individual experience. At the same time, it is difficult to deny the phenomenal nature of the relational aspects of consciousness, such as seeing the object place itself in the foreground and the background extend behind it.

If we limit ourselves to vision, some of the most frequently described qualia are the ones that refer to colors. Scholars refer to the redness of red, using terminology that is different from the one of common sense and referring to a visual experience that is distant from the usual ones. Interestingly, scholars do not refer to the way we see a face, which is much closer to the reality of conscious vision, and which is used as a prototype of conscious experience in many experiments on neural correlates of consciousness (Koch et al., 2016). This is probably because it would be much more difficult to describe the phenomenal experience of a face in non-relational terms.

Dennett (1988) defines qualia as the ways things seem to us. As an example, he cites the way we see a glass of milk at sunset. According to Dennett, “the particular, personal, subjective visual quality of the glass of milk at sunset is the *quale* of your visual experience at the moment.” However, it is very difficult to have this kind of experience and describe it in the absence of its internal relations: the whiteness and liquidity of milk, the fact that the milk is contained in the glass, the convexity and transparency of the glass, the table on which the glass of milk is standing, the sun next to the glass that disappears over the horizon, the particular light of sunset that affects the way the glass looks, the feeling that this vision can arouse, and our state of mind when we see the glass. What would this experience be without these relations? Would it be an experience in an absolute sense? Is it possible to really separate the elements that, in relation to each other, make up our phenomenal reality from the way they seem to us? Are we assuming that there is a conscious quale of the vision of the glass of milk at sunset that is associated with the non-conscious vision of the glass of milk at sunset?

Or are we assuming that qualia give to a perceptual state the particular qualities that would make it phenomenal, whereby the phenomenal character would be determined in the relation between qualia and perceptual state? In other words, “qualia … are properties of sensations and perceptual states, namely the properties that give them their qualitative or phenomenal character—those that determine ‘what it is like’ to have them” (Shoemaker, 1991). This could mean that, in order to be conscious, the vision of an object must have particular qualities. But the conscious nature of perception is either an expression of the set of relations existing between the components of the field—without our being able to confidently assign a particular status to any of them—or we must assume that something similar happens to when the magic dust from the wand of Cinderella’s fairy godmother turns the pumpkin into a carriage.

The intrinsic nature of qualia can be traced to the supposed simplicity of some of them, such as the ones related to color. But this misunderstanding stems from a phenomenal simplicity of the perception of a color which, in fact, is not so simple. Let us try to replace red with black in a black-and-white world, made up of black, white and a range of grays. It is a simpler world, but it is to all intents and purposes a phenomenal world. In a black-and-white world, it becomes much more difficult to speak of the blackness of black as an intrinsic element. Dark gray is phenomenally dark gray because it differs from the white background more than light gray and less than black. Black is black because it equals black and differs from white more than any shade of gray. It is hard to imagine that this does not apply to a color like red that is immersed in a more complex range of relations, including, in addition to the light–dark dimension, saturation and relation to other colors.

In this sense, a certain shade is necessarily related to something that is outside the field of the stimulus. Perception of so-called elemental qualities implies the involvement of memory in the conscious field, as Edelman (2001) eloquently expressed with the concept of *remembered present*. Perceiving a color implies similarities and differences with reference patterns that cannot derive solely from the present stimulus and that must consciously manifest themselves somehow, e.g., “in the background.” In other words, not only the premises of the perceived quality, but also the perceived quality itself, in the way it is perceived, imply the involvement of elements that are not present in the stimulus. One of the properties of qualia, which gives them their universal character, is precisely the fact of being recognized from one experience to another (Lewis, 1929).

Briefly, it is more correct to speak of *qualitative aspects as components of experience* rather than qualia. They cannot be analyzed independently of the experiential field to which they belong. If we consider qualitative aspects taken in isolation as fully representative of experience, we distort their phenomenal essence. It is an operation that creates a phenomenal artifact (de Laguna, 1916).

If, on the contrary, we admit that the qualitative aspects of experience cannot be extrapolated from the context to which they belong without undermining their phenomenal nature, an important consequence is that they are necessarily relational. Being relational is an integral part of the nature of what is qualitative. Unless we assume that *the entire* field of experience is something intrinsic, monadic, and nonrelational, the object of phenomenal analysis can only be the field of experience in its totality and in its internal relations.

## The simplest aspects of consciousness should be researched in perception

Another consequence of considering qualitative aspects of experience as relational is that we will not necessarily focus primarily on the qualitative aspects of what we might call the classical qualia. Although it can occur in the simplest forms of consciousness, a qualitative feel is something that characterizes a conscious experience but is not identified with it. None of us perceives the quale of green, of sweetness, of pain *alone*. We perceive *something* green, something sweet, we perceive pain in a part of the body and therefore in relation to it. We cannot help but perceive these sensations in relation to *something*. There is no evidence that by eliminating what green belongs to, it would retain those phenomenal properties or that it would retain phenomenal properties in general. Also in common usage, in addition to having a positive or negative connotation, a quality is a characteristic or feature that someone or something has (Encyclopædia Britannica, 2023).

Moreover, if the quality is inevitably the quality of something, *this something is in turn always in relation to a background*. In other words, a phenomenal quality cannot but belong to something, and this something cannot but belong to a background. Green belongs to the leaf, pain to the knee. In turn, the green leaf is on the tree, the painful knee is in the leg. As Merleau-Ponty (1945) points out, “at the outset of the study of perception, we find in language the notion of sensation, which seems immediate and obvious: I have a sensation of redness, of blueness, of hot or cold. It will, however, be seen that nothing could in fact be more confused, and that because they accepted it readily, traditional analyses missed the phenomenon of perception … When Gestalt theory informs us that a figure on a background is the simplest sense-given available to us, we reply that this is not a contingent characteristic of factual perception, which leaves us free, in an ideal analysis, to bring in the notion of impressions. It is the very definition of the phenomenon of perception, that without which a phenomenon cannot be said to be perception at all. The perceptual ‘something’ is always in the middle of something else, it always forms part of a ‘field’… The pure impression is, therefore, not only undiscoverable, but also imperceptible and so inconceivable as an instant of perception.” A qualitative feel, insofar as it relates to a perceptual “something” that belongs to a “field,” merely adds a sensory aspect to this dyad.

There is a philosophical tradition that tends to attribute the primitive aspects of experience to sensation. According to Reid (1764/1997), if sensation is a simple, subjective datum, perception is a complex cognitive act that actively unifies a set of sensations by ascribing them to an object. It is widely believed that the most relevant aspect of perception is the extraction of relevant information from sensation: detecting, identifying, recognizing (Fesce, 2023). The idea that sensations precede perception (Gärdenfors, 2006) has been somewhat reframed by the attribution of the simplest forms of phenomenal experience to qualia and raw feelings. However, perception is not a more complex and organized form of sensation. The formation of the object is the *sine qua non* for the occurrence of experience. In my view, sensations can only occur in a perceptual context that is, *ab initio*, multisensory (Bennett and Hill, 2014; Bayne and Spence, 2015; O’Callaghan, 2015) and in which sensations are in a way dependent on perceptual aspects. In other words, they can only occur within a conscious perceptual experience (Hardin, 1992). On this basis, rather than with classic qualia and simple sensations, basic consciousness might coincide with perception and the qualitative aspects associated with it.

It could be argued that our experience does not necessarily refer to an object. Even without making reference to the Eastern disciplines (Srinivasan, 2020), it is enough to close our eyes to experience darkness. But in these cases we cannot help but experience our body: if we focus on the visual experience, our body will act as a background to the darkness we perceive and will in turn be perceived in the background of the perceptual space in which our body is located (Jerath et al., 2015). The conscious perception of light and dark, which is identified as one of the simplest things we can perceive (Edelman and Tononi, 2000), is only possible at a level comparable to that of the perception of an object.

If we keep in mind that, in the classical sense of the term, quality is such in relation to a reference pattern existing in memory, that it is in relation to an object, and that the object is in relation to a contrasting background, it is clear that the simplest aspects of phenomenal experience can be detected most easily in a simple figure and thus in early vision. In this sense, early vision is what gestaltists call “primary vision,” which occurs even before object recognition (Kanizsa, 1979, 1980, 1991). Kanizsa (1980) states that “visual perception is a complex cognitive activity, in which it is possible to distinguish at least two levels or moments: the moment of the formation of the visual object, i.e., the primary process by which sensory input is organized and segmented, and a secondary process that includes the more properly intellectual operations of categorization, signification, and interpretation that the mind performs on the results of primary segmentation.” So, I am referring to early vision as the simplest form of visual experience, related to perceptual organization. It does not involve interpretation or other strictly cognitive processing of visual information.

The figure/background organization is often listed among the properties of consciousness, with similar but not entirely overlapping meanings such as foreground/background, situation, figure/background, center/periphery, selection or choice (James, 1890; Zeman, 2001; Edelman, 2003; Searle, 2004; Northoff et al., 2023). After all, vision—which I will address here in its phenomenal aspects—is the preferred field of investigation of consciousness for many authors (Koch, 2004; Jerath et al., 2015; Lamme, 2020; Ludwig, 2023). It is worth noting that for gestaltists perception is not preceded by sensation but is a primary and immediate process. Structured wholes or Gestalts, rather than sensations, are the primary units of mental life (Wagemans et al., 2012). According to Lamme (2020), perceptual organization is the visual function that is central to understanding the transition from unconscious to conscious seeing. Processes of grouping and figure-ground segregation depend strongly on the stimulus that is evoking these operations being consciously perceived.

It could be argued that, by investigating the principles that determine the grouping or the choice of a region of the field as an object rather than as a background, the Gestalt approach somewhat circumvents the hard problem, since it limits itself to the so-called “functional” aspects of perceptual organization (Lamme, 2010, 2020). However, it should be pointed out that the perception of a figure against a background cannot be equated with the mere result of an operation like the assignment of borders, to which cognitive science attributes the choice of the object (Williford and von der Heydt, 2013). Ever since Rubin’s first descriptions, it has been clear that a figure seen against the background of something has purely phenomenal characteristics. The figure has an object-like character, and there is a tendency to see the figure as positioned in front, and the ground at a further depth plane and continuing to extend behind the figure. Furthermore, the border separating the two segments is perceived as delineating the figure’s shape as its contour, whereas it is irrelevant to the shape of the ground (Todorovic, 2008).

These characteristics are not taken into account in identifying the basic phenomenal aspects of consciousness. However, they are no less qualitative than the redness of red and the painfulness of pain. Moreover, in the visual field there are not only the figure and the background. A visual object is not such if, in addition to differentiating itself from the background, it does not have an inhomogeneity that underlies its details, its constituent parts and its surface texture. Secondly, in addition to the object and background there are secondary objects and backgrounds, elements that come together to form Gestalts, and so on. Likewise, being an object, a detail, a Gestalt or a secondary object involves attributing a certain phenomenal quality to that part of the field.

The qualities of the field components that result from perceptual organization appear even simpler than the ones usually identified with qualia, with raw feelings and seemingly elementary aspects of phenomenal experience: redness, sweetness, painfulness, roundness, distinction between light and dark. In contrast to classical qualitative aspects, the quality related to being an object can be derived exclusively from features present in the stimulus. There is no need to bring up anything from memory to see an object against the background of something. Although there is no unanimous agreement on this point, it can be argued that in most cases the relation of the object to the background depends on autochthonous factors, that is, on factors that are all in the stimulus, thereby they do not depend on previous knowledge, expectancies, voluntary sets, intentions of the observer (Luccio, 2011).

The Gestalt approach is for all intents and purposes a phenomenological approach. However, in studying perceptual organization, it has addressed very simple aspects of conscious experience. The perceptual field is made up of figure and background, main objects and secondary objects, clear components and other less clear components. One reason why it is difficult to conceive of the perceptual field in its entirety is the progressive fading of its components. However, this is an aspect that is part of consciousness and that cannot be ignored. It is therefore necessary to formulate a conception of experience that includes its fading. One problem lies in the fact that perceptibility declines progressively, with no clear boundary between what we see clearly and what we do not see at all. It is worth noting that in very simple stimulus conditions, as in many of those studied by Gestaltists, we can sufficiently perceive all the relationships in the field, partially overcoming this difficulty.

Galus and Starzyk (2020) and Galus (2023a,b) propose the Reductive Model of the Conscious Mind. It is based on the distinction among different aspects of consciousness served by independent neural processes. According to the authors, attempts to define the phenomenon of consciousness have encountered difficulties. They seemed insurmountable because they strived to explain a multifaceted phenomenon, realized by completely different neural, biophysical, and behavioral phenomena, using one definition, one process or property of matter. The basic structure of consciousness includes three main aspects: Perceptual Consciousness, Executive Consciousness, and Reporting Consciousness. This complex view includes perceptions, the manipulation of the world and of objects, the sensations we derive from this manipulation, emotions, interoception of states of the organism that deviate from a condition of homeostasis. Embodiment requires having a body equipped with senses of external and internal signals reporting on the state of the environment and the state of homeostasis. This body must also be able to respond to detected signals from the environment and its own body.

It is worth noting the hypothesis of how secondary perception can visualize thoughts as well as imagery, memories, and dreams. As Galus (2023a) underlines, “the more important aspect of secondary signal transmission up-down is the dramatic increase in the ability to learn and analyze situations quickly. Thanks to the visualization of one’s thoughts, it was possible not only to react directly to sensory stimuli but also to imagine the sequence of actions and plan the reactions optimally. Moreover, it is less about the logical analysis of possible responses and making appropriate decisions but about the idea of how one’s body functions, muscle tension, the position of the limbs, and the dynamics of movements.”

The scope of my paper is much more limited. I focus on a simpler level. I refer neither to classically defined qualia, nor to interoception. Of course, emotions and qualia of internal states play a fundamental role for the mental states aimed at maintaining homeostasis. However, as we have seen with regard to the phenomenal nature of the perception of object and background, even a simple visual perception is conscious and must be explained and justified as such. Following the distinction of Galus and Starzyk (2020), I think that direct perception can be conscious even if it is not accompanied by phenomenal feelings.

The role of the relationship between subject and object in basic consciousness remains to be clarified. The question is whether this apparently obvious role (Searle, 2004; Damasio, 2010; Damasio and Damasio, 2022) is a fundamental aspect of PC. As phenomenologists argue (Gallagher and Zahavi, 2008), even in the absence of self-consciousness in the full sense of the term, consciousness would be characterized by pre-reflective self-consciousness, which is involved in having experiences as one’s own and can be construed as a kind of low-level self-consciousness (Flanagan, 1992). In a similar sense, Kriegel (2004) speaks of peripheral self-consciousness.

If the basic aspect of consciousness is perception in its simplest forms, it appears less intimately linked to subjectivity than sensations. It is certainly true that our conscious experiences are subjective. However, it is one thing to take an interest in the world around us; it is another to observe ourselves as we observe the world. It is one thing to have an egocentric perspective; it is another to have an allocentric perspective, such as when we look at a map. Sensations—such as heat or pain—that directly concern the subject and its relations to the outside world are one thing; “distal” features of the outside world that are such because of the relations between the elements that make it up—such as the roundness of an object or the number of trees in the forest in front of us—are another. When we turn our attention to the outside world, our conscious experiences are not characterized by introspective awareness (Seager, 2002). When we become absorbed in some intense perceptual task, we are vividly conscious but, often, we may lose the sense of self (Tononi and Koch, 2008).

If we hypothetically eliminated the subjective component of consciousness, the phenomenal problem of vision—about why a red triangle appears as such and it is not just a configuration eliciting a response—would still remain unsolved. The fact that a red triangle appears *to us* cannot be the only element accounting for its appearance and for its phenomenal ontology (Forti, 2009). It is therefore possible to temporarily set aside the problem of subjectivity. As Merleau-Ponty (1945) points out, “it is the very notion of the immediate which is transformed: henceforth the immediate is no longer the impression, the object which is one with the subject, but the meaning, the structure, the spontaneous arrangement of parts.”

In my view, early vision can represent a form of experience that, by allowing subjectivity to be temporarily put in abeyance, provides a pathway to consciousness that may facilitate the formulation of third-person theoretical constructs. Experimental situations in which gestalt laws are tested represent experiences that feature characteristics of phenomena observable in the third person perspective. Or, at least, the role of the subject can be considered irrelevant. In these situations, what we see seems to depend phenomenally on the relationships between the components of the field rather than on the relationships between object and percipient subject. Most Gestalt laws concern the organization of conscious vision. They are based exclusively on the relations existing in the perceptual field, starting with the relation of the object to the background (Luccio, 2011). Of course, vision necessarily implies a point of view, but it is the same with many recording and measuring instruments. Moreover, perception can be considered a public mode of observation. In this sense, visual perception has aspects in common with the scientific approach, of which, through observation of the world around us, it is the basis (Gallagher and Zahavi, 2008).

## The explanandum is a unitary whole of qualities

What do we find if we analyze the simplest forms of visual perceptual experience? The first observation might be in some respects obvious and in others questionable: the simplest aspects of consciousness can be seen in the perception of a simple figure against the background of something (Merleau-Ponty, 1945). Unlike classical qualia, a figure has a clear relation to the background, which is essential for the perception to have the phenomenal characteristics that are well known to us. The “quale” of the object can only be perceived or conceived in relation to the “quale” of the background.

But not only the figure against the background of something is relational. We have seen that all qualitative aspects of consciousness are relational. These aspects include the ones that are usually attributed to qualia, whereby the quality is such in relation to a reference pattern in memory and is in relation to an object that is in turn in relation to a contrasting background. If we simply examine the relations existing in early perception, the relational aspect is even more evident. Any content can only have phenomenal characteristics in relation to other contents or aspects of the perceptual field, starting from the object and background. Being the main object implies at least a background, other objects over which it prevails, as well as the details and parts of which it is made up.

Another fundamental aspect of phenomenal experience, related to the previous one, is the unity we experience in all perceptions. Since Descartes and Kant, unity has been considered by almost all authors to be among the fundamental characteristics of consciousness. Often, the attribution of unity to consciousness has implied a monadic conception of consciousness. It should be noted that identifying consciousness with a simple and intrinsic unity is not the exclusive prerogative of classical qualia. In fact, it includes most qualitative conceptions—or conceptions referable to the idea of “what it is like to be”—insofar as reference is made to something that does not appear to be analyzable in its internal structure.

In my opinion, it is preferable to adopt a conception—like the gestalt—whereby unity is not monadic but is such through the interdependence of the parts that make up the field of experience (Kanizsa, 1980; Wagemans et al., 2012; Tononi and Koch, 2015). Unity is clearly found in the visual experiences described by gestaltists, starting from the relationship between part and whole. In a simple perceptual situation, the relations between the elements of the field are characterized by mutual dependence, in the sense that each component of the field is such in function of the others, e.g., object-background, gestalt-constituent elements, object-detail, main object-secondary object. Interdependence seems to involve multiple elements of the field at the same time. A detail could not be perceived as part of an object if at the same time the object were not perceived as belonging to a background. We thus move from a monadic conception of consciousness to a conception whereby the qualitative aspects of consciousness are necessarily manifold and at the same time closely related to each other. The phenomenal analysis of perceptual experience highlights that its qualitative aspects are relational and that consciousness appears to us as unitary through the mutual dependence of these relations. Consequently, we can say that the explanandum is a *unitary set of qualities*, i.e., a set of qualities closely dependent on each other, which we can find in its simplest forms in early vision. Such an explanandum may appear insufficient, but it certainly cannot be reduced to something that does not include these features taken together. This conception is clearly different from the mosaic of qualia, which entails a mere combination of different qualities (Jansen, 2017). The relationship between the various qualitative aspects is something more complex. It entails relationships of interdependence and on different hierarchical levels—not only between objects, but also between contiguous regions.

Above I stated that unity *per se* is not specific to consciousness, as it could belong to many non-conscious organizations, and that, if a property such as unity undoubtedly applies to consciousness, then we should understand how the unity that manifests itself on the phenomenal level differs from other forms of unity (Wiese, 2017). The concept of a unitary set of qualities is well suited to this statement, as unity concerns specific properties of consciousness such as the qualitative aspects. In this sense, the qualities that characterize consciousness are necessarily interdependent parts of a whole that encompasses the entire field. The co-presence of the qualitative aspect and the unity aspect is thus crucial in identifying the explanandum of consciousness.

Unlike Gestaltists and other authors (Tononi and Koch, 2015), this totality should not be identified in the object as a structured whole, but in the total field of experience, which includes background, fringe parts and progressively fading components. We often consider only the most salient contents of consciousness, disregarding the progressively fading field and ignoring other components even when they are sufficiently perceptible. If we do not limit ourselves to the main object, its main features, and the gestalts present in the field, but we also take into account elements such as the background, secondary objects, parts of an object, components of a gestalt, and less important elements, the unitary set of qualities that we identify in a phenomenal analysis of early perception becomes progressively evanescent. The difficulty of dealing with such situations can be partly overcome by limiting ourselves to the simplicity of many stimulus situations analyzed by Gestaltists, in which the progressive fading of the perceptual field is negligible.

## Discussion: in search of the unitary structure of consciousness

At first glance, one might think that identifying the explanandum in a unitary set of qualities is equivalent to identifying the structure of consciousness, at least in such elementary forms as early vision. But things are not so simple. I started from the need to analyze in structural terms qualia or, rather, the qualitative aspects of consciousness. The analysis of the simplest forms of perceptual consciousness led us to point out that these qualitative aspects are not only relational, but also form a unitary whole. Thus, the existence of a unitary set of qualities does not allow us to limit ourselves to analyzing in structural terms a single quality. We must also explain their relational nature, the way their relations form a unitary whole and their interdependence in perceptual organization. On the one hand, this explanation may seem more difficult. On the other hand, we can assume that quality and interdependence are somehow related, at least in early vision.

Jones and Hunt (2023) approach this issue in a similar way, but do not challenge the phenomenal reality of qualia. According to these authors, the main problems in neuroscience’s accounts of qualia seem to fit into three categories: the coding/correlation problem, the qualia-integration problem, and the hard problem. In my view, these are not three distinct questions, even though they are interrelated; they constitute a single fundamental question, which is to explain the unitary set of qualities encountered in early perception.

With regard to the unity of visual experience, it is not sufficient to say that the various qualitative aspects of consciousness are perceived as interdependent. The perceived interdependence does not explain the qualities of perceptual experience, but neither does it explain how these qualities form a unitary whole. Saying that the explanandum is a unitary whole of qualities is not the same as identifying the structure of the consciousness, i.e., how that whole is organized into a unitary whole. Consciousness should have a structure that justifies such unity.

Therefore, Loorits’ argument that consciousness should have a structure must be completed by stating that the structure of consciousness should have that unitary character that is typical of consciousness. The goal is to look not for a series of separate structural aspects, but for a unitary structure. We must ask ourselves whether the relational aspects we identify in experience are compatible with the unity we feel in all perceptual experiences. We cannot separate these aspects. It is neither sufficient to identify on its own the unity we all feel in our experience, nor to identify relational or structural aspects that do not ensure unity by themselves. In a way, a phenomenal analysis goes over the two poles of conscious experience: its being composite, in that it is made up of multiple qualitatively characterized contents or phenomenal distinctions, and at the same time unitary, so much so that, through qualia, it recalls the idea of a monad. How is it possible to reconcile these two poles?

The unity manifested through the interdependence of the parts of the field of experience can be interpreted as a form of integration. Tononi and Koch (2015) propose the Integrated Information Theory (IIT) and list structure (composition) and unity (integration) among the properties of consciousness. In this sense, as a result of the interdependence of phenomenal distinctions, integration is phenomenal evidence rather than a theory. Historically, this has been clear to many authors who have tried to define consciousness (Brogaard et al., 2021; Hirschhorn et al., 2021; Solms, 2021). The problem is to explain how integration, as manifested in conscious experience, can come about. The IIT postulates an organization of the neuronal substrate characterized by complexity and by the presence of high levels of integration and differentiation. This proposal appears to be an almost tautological and overly general explanation to justify the *particular kind* of integration that we observe in experience. Life also involves a complex organization of organic molecules, but postulating a high level of complexity is not sufficient to explain it. Moreover, the IIT does not address the specific qualitative aspect (Cooke, 2021), so it is precisely the qualitative aspects that are integrated into experience. Even if the IIT proposes an explanation for the qualitative aspect (Tononi, 2008), it does not correlate it with the integration that occurs in the perceptual field. By not including an explanation of the qualitative aspects and their relations, a complex system such as the one postulated by the proponents of the IIT may belong to non-conscious organizations.

Moreover, structure should be constitutive, not just reflecting relations existing in the stimulus field. We should identify a structure that is not contingent, but constitutive of each experience and somewhat independent of the type of stimuli (Buzsáki, 2007; Bayne et al., 2016; Smith, 2018; Kent and Wittmann, 2021; Northoff and Zilio, 2022). Many structural aspects highlighted in the literature seem to reflect the organization in the apparent reality of specific contents rather than the internal structure of the conscious field. Of course, we can assume that conscious structure allows us to capture structural aspects of the reality around us, so the ability to capture a structure present in external reality may also be an expression of conscious structure.

A unitary structure can be identified in a simple relationship between figure and background and in their interdependence. The coherence and unity of what we perceive cannot be separated from its belonging to the background: “the background, which need never have been made determinate, affects the appearance of what is determinate by letting it appear as unified, bounded figure” (Dreyfus, 1992). It should be emphasized that this is a phenomenal unitary structure, in that object and background have qualitative characteristics that appear as a function of each other. In essence, there is a unitary structure in the simplest manifestation of consciousness, a phenomenal object in the form of a simple figure. This structure appears constitutive and non-contingent, because we cannot perceive the object without the background. It is constitutive because without this relationship there is no consciousness—even though this relationship reflects a fundamental aspect in the surrounding reality, i.e., the fact that as a rule the world is made up of objects in a space.

However, if we analyze images that are just a little more complex, a unitary structure becomes more difficult to detect. Unity, which manifests itself through the interdependence of the parts, remains perceptible, but we cannot identify the structure underpinning it. It is possible to identify relational qualitative aspects, but they do not seem able to provide phenomenal unity. In their comprehensive approach, gestaltists postulate the unity of the field, but they do not explain it (Wagemans et al., 2012). The various Gestalt laws explain in heterogeneous ways different forms of grouping and the figure-background organization, but not the unity of the perceptual field.

Faced with the heterogeneity of relations between the parts, the apparent unity of perceptual experience leads us to wonder *how* these different relations constitute a unitary whole. Object, background, gestalt, detail, secondary objects are all expressions of the relationship with something else, but, at first glance, they do not allow us to understand *how* they constitute a unitary whole. We might say that the various phenomenal qualities are not all on the same plane. In a perceptual experience the main object stands in the foreground. Other qualities are associated with it in a subordinate way; others are associated with such qualities, and so on, until they completely fade away. However, not even conceiving the various phenomenal qualities as a set of progressively fading hierarchical relationships justifies the apparent unity of the field. Indeed, the phenomenally subordinate relationship of the qualities associated with the main object is not limited to the background’s secondary role, but it includes such heterogeneous relationships as the ones involving the secondary objects, parts, details, and elements that form a gestalt. Why do objects, backgrounds, gestalts and details appear as they appear and at the same time are part of a unitary experience?

If we focus on a more complex image than a figure against a homogeneous background, it is not enough to say that on the table there are a bottle, two plates and some glasses, that a picture hangs on the wall, and that we perceive these objects as a unitary whole. There is a gap between the unity we perceive and the possibility of identifying the structure underlying it through relationships that make it possible. We cannot identify the structure that provides the unity we experience and perceive even when the composition of an image seems random. We can put random elements into a visual field (Kanizsa, 1980) and the image will retain its own unity. Thus, unity is not merely contingent.

At a preliminary phenomenal analysis, the problem of the unitary structure of consciousness seems without solution. The fact that we perceive the experience as unitary and perceive the various qualitative aspects as interdependent seems to be a kind of mystery for which we cannot find an explanation, either in brain organization or in experience itself. Phenomenal appearance alone does not seem sufficient to identify a unitary structure of consciousness.

This paper has arguably achieved the goal of identifying an explanandum in terms that can be useful for research, but it has not achieved the goal of identifying the unitary structure of consciousness. The unitary set of qualities that I have identified as the explanandum of consciousness is not a real structure, let alone a unitary structure. While it is a unitary set of qualities, it neither tells us what the structure of seemingly non-structural aspects like the qualities of object and background is, nor does it identify the unitary character of that structure. However, this does not mean going back to the search for the physical substrate that has proven to be dead-end. Elements of “closeness” with the structural aspects of consciousness can be found in appearance itself. The results of the analysis of the simplest forms of perceptual experience, with the presence of closely interdependent qualitative components that form a unitary whole prompt us to go beyond the mere phenomenal appearance, using the components of experience also as possible explanans.

One of the most obvious explananda is appearance, which is nothing else than the etymological meaning of consciousness as a phenomenal entity. In the simplest sense, it implies the possibility of something being perceived consciously. However, it is the very appearance and the way it is structured in perceptual experience that makes us think that the perceptive field contains within itself parts responsible for the appearance, yet they remain imperceptible. Other components of experience that could constitute a possible explanans are generally neglected phenomenal aspects like overlapping of the contents of the field and surroundedness. Surroundedness is a relationship whereby a region is surrounded by or surrounds a contrasting region, and it has a broader meaning than the one we assign to the figure-ground relationship. I will examine the possibility of going beyond the mere phenomenal appearance elsewhere, hypothesizing that the structure of consciousness is somehow conscious, although “hidden” from consciousness itself. Such a structure might provide a kind of link that can bridge—or at least reduce—the explanatory gap between experience and brain processes and thus help solve the hard problem.

## Data availability statement

The original contributions presented in the study are included in the article/supplementary material, further inquiries can be directed to the corresponding author.

## Author contributions

BF: Writing – original draft.

## References

[ref1] Baars B. J. (1997). In the theatre of consciousness: global workspace theory, a rigorous scientific theory of consciousness. J. Conscious. Stud. 4, 292–309.

[ref2] Bayne T. (2010). The unity of consciousness. Oxford: Oxford University Press.

[ref3] Bayne T. Hohwy J. Owen A. M. (2016). Are there levels of consciousness? Trends Cogn. Sci. 20, 405–413. doi: 10.1016/j.tics.2016.03.009 27101880

[ref4] Bayne T. Spence C. (2015). “Multisensory perception” in The Oxford handbook of philosophy of perception. ed. Matthen M. (Oxford: University Press), 603–620.

[ref5] Bennett D. Hill C. (2014). Sensory integration and the Unity of consciousness. Cambridge, MA MIT Press.

[ref6] Block N. (1995). On a confusion about a function of consciousness. Brain Behav. Sci. 18, 227–247. doi: 10.1017/S0140525X00038188

[ref7] Block N. (2005). Two neural correlates of consciousness. Trends Cogn. Sci. 9, 46–52. doi: 10.1016/j.tics.2004.12.006 15668096

[ref9] Boly M. Massimini M. Tsuchiya N. Postle B. R. Koch C. Tononi G. (2017). Are the neural correlates of consciousness in the front or in the Back of the cerebral cortex? Clinical and neuroimaging evidence. J. Neurosci. 37, 9603–9613. doi: 10.1523/JNEUROSCI.3218-16.2017, PMID: 28978697 PMC5628406

[ref10] Bond E. (2023). The contribution of coherence field theory to a model of consciousness: electric currents, EM fields, and EM radiation in the brain. Front. Hum. Neurosci. 16:1020105. doi: 10.3389/fnhum.2022.1020105, PMID: 36760225 PMC9903675

[ref11] Brogaard B. Gatzia D. E. Chomanski B. (2021). Consciousness and information integration. Synthese 198, 763–792. doi: 10.1007/s11229-020-02613-3

[ref12] Brown R. Lau H. LeDoux J. E. (2019). Understanding the higher-order approach to consciousness. Trends Cogn. Sci. 23, 754–768. doi: 10.1016/j.tics.2019.06.009 31375408

[ref13] Buzsáki G. (2007). The structure of consciousness. Nature 446:267. doi: 10.1038/446267a 17361165

[ref14] Calabrò R. S. Cacciola A. Bramanti P. Milardi D. (2015). Neural correlates of consciousness: what we know and what we have to learn! Neurol. Sci. 36, 505–513. doi: 10.1007/s10072-015-2072-x, PMID: 25588680

[ref15] Chalmers D. J. (1995). Facing up to the problem of consciousness. J. Conscious. Stud. 2, 200–219.

[ref16] Chalmers D. (2007). “The hard problem of consciousness” in The Blackwell companion to consciousness. eds. Velmans M. Schneider S. (Chichester, UK: Blackwell), 32–42.

[ref17] Cooke J. E. (2021). What is consciousness? Integrated information vs. inference. Entropy 23:1032. doi: 10.3390/e23081032, PMID: 34441172 PMC8391140

[ref18] Crick F. Koch C. (1998). Consciousness and neuroscience. Cereb. Cortex 8, 97–107. doi: 10.1093/cercor/8.2.97 9542889

[ref19] Damasio A. (2010). Self comes to mind: Constructing the conscious brain. New York: Pantheon Books.

[ref20] Damasio A. Damasio H. (2022). Homeostatic feelings and the biology of consciousness. Brain 145, 2231–2235. doi: 10.1093/brain/awac194, PMID: 35640272

[ref21] de Laguna G. A. (1916). Sensation and perception. I: the genetic relationship. J. Philos. Psychol. Sci. Methods 13, 533–547. doi: 10.2307/2012564

[ref22] de Leon D. (2001). The qualities of qualia. Commun. Cogn 34, 121–138.

[ref23] Dehaene S. (2014). Consciousness and the brain: Deciphering how the brain codes our thoughts. New York: Viking Penguin.

[ref24] Dehaene S. Kerszberg M. Changeux J. P. (1998). A neuronal model of a global workspace in effortful cognitive tasks. Proc. Natl. Acad. Sci. USA 95, 14529–14534. doi: 10.1073/pnas.95.24.14529 9826734 PMC24407

[ref25] Dennett D. C. (1988). “Quining qualia” in Consciousness in contemporary science. eds. Marcel A. J. Bisiach E. (Oxford: Clarendon Press), 42–77.

[ref26] DiCarlo J. J. Zoccolan D. Rust N. C. (2012). How does the brain solve visual object recognition? Neuron 73, 415–434. doi: 10.1016/j.neuron.2012.01.010, PMID: 22325196 PMC3306444

[ref27] Doesburg S. M. Green J. J. McDonald J. J. Ward L. M. (2009). Rhythms of consciousness: binocular rivalry reveals large-scale oscillatory network dynamics mediating visual perception. PLoS One 4:e6142. doi: 10.1371/journal.pone.0006142, PMID: 19582165 PMC2702101

[ref28] Dreyfus H. L. (1992). What computers still Can’T do: A critique of artificial reason. Cambridge, MA: MIT Press.

[ref29] Edelman G. (2001). Consciousness: the remembered present. Ann. N. Y. Acad. Sci. 929, 111–122. doi: 10.1111/j.1749-6632.2001.tb05711.x 11349421

[ref30] Edelman G. M. (2003). Naturalizing consciousness: a theoretical framework. Proc. Natl. Acad. Sci. USA 100, 5520–5524. doi: 10.1073/pnas.0931349100, PMID: 12702758 PMC154377

[ref31] Edelman G. M. Tononi G. (2000). A universe of consciousness: How matter becomes imagination. New York: Basic Books.

[ref32] Encyclopædia Britannica (2023). Quality. Available at: https://www.britannica.com/dictionar/quality (Accessed October 11, 2023).

[ref33] Feldman J. (2013). The neural binding problem(s). Cogn. Neurodyn. 7, 1–11. doi: 10.1007/s11571-012-9219-8, PMID: 24427186 PMC3538094

[ref34] Fesce R. (2023). Imagination: the dawn of consciousness: fighting some misconceptions in the discussion about consciousness. Physiol. Behav. 259:114035. doi: 10.1016/j.physbeh.2022.114035, PMID: 36403782

[ref35] Flanagan O. J. (1992). Consciousness reconsidered. Cambridge, MA: MIT Press.

[ref36] Forti B. (2009). How could phenomenal consciousness be involved in mental function? New Ideas Psychol. 27, 312–325. doi: 10.1016/j.newideapsych.2008.10.001

[ref37] Forti B. (2021). Subjective experiences matter. What do we know about consciousness? Evid. Based Psychiatr. Care 7, 158–169. doi: 10.36180/2421-4469-2021-25

[ref38] Friedman G. Turk K. W. Budson A. E. (2023). The current of consciousness: neural correlates and clinical aspects. Curr. Neurol. Neurosci. Rep. 23, 345–352. doi: 10.1007/s11910-023-01276-0, PMID: 37303019 PMC10287796

[ref39] Gallagher S. Zahavi D. (2008). The phenomenological mind. New York: Routledge

[ref40] Gallotto S. Sack A. T. Schuhmann T. de Graaf T. A. (2017). Oscillatory correlates of visual consciousness. Front. Psychol. 8:1147. doi: 10.3389/fpsyg.2017.01147, PMID: 28736543 PMC5500655

[ref41] Galus W. (2023a). Different aspects of consciousness explained by distinct biophysical processes. J. Theor. Philos. Psychol. doi: 10.1037/teo0000236

[ref42] Galus W. (2023b). Mind–brain identity theory confirmed? Cogn. Neurodyn. 17, 1–21. doi: 10.1007/s11571-023-09992-6 PMC11297862 39104703

[ref43] Galus W. Starzyk J. (2020). Reductive model of the conscious mind. Hershey, PA: IGI Global isbn:13: 9781799856535.

[ref44] Gärdenfors P. (2006). How Homo became sapiens: On the evolution of thinking. Oxford: Oxford Academic (Accessed October 13, 2023).

[ref45] Ghosh H. (2020). “Early vision” in Computational models for cognitive vision. (New York: IEEE), 9–24. doi: 10.1002/9781119527886.ch2

[ref46] Hameroff S. Penrose R. (2014). Consciousness in the universe: a review of the 'Orch OR' theory. Phys Life Rev 11, 39–78. doi: 10.1016/j.plrev.2013.08.002 24070914

[ref47] Hardin C. L. (1992). “Physiology, phenomenology, and Spinoza’s true colors” in Emergence or reduction?: Essays on the prospects of nonreductive physicalism. eds. Beckermann A. Flohr H. Kim J. (Berlin: De Gruyter), 201–219.

[ref48] Haun A. Tononi G. (2019). Why does space feel the way it does? Towards a principled account of spatial experience. Entropy 21:1160. doi: 10.3390/e21121160

[ref49] He B. J. (2023). Towards a pluralistic neurobiological understanding of consciousness. Trends Cogn. Sci. 27, 420–432. doi: 10.1016/j.tics.2023.02.001, PMID: 36842851 PMC10101889

[ref50] Hirschhorn R. Kahane O. Gur-Arie I. Faivre N. Mudrik L. (2021). Windows of integration hypothesis revisited. Front. Hum. Neurosci. 14:617187. doi: 10.3389/fnhum.2020.617187, PMID: 33519404 PMC7840615

[ref51] Humphrey N. (2006). Seeing red: A study in consciousness. Harvard: Harvard University Press.

[ref52] James W. (1890). The principles of psychology. London: MacMillan.

[ref53] Jansen F. (2017). The hard problem of consciousness from a bio-psychological perspective. Philos. Stud. 7, 579–594. doi: 10.17265/2159-5313/2017.11.001

[ref54] Jerath R. Crawford M. W. (2014). Neural correlates of visuospatial consciousness in 3D default space: insights from contralateral neglect syndrome. Conscious. Cogn. 28, 81–93. doi: 10.1016/j.concog.2014.06.008, PMID: 25049208

[ref55] Jerath R. Crawford M. W. Barnes V. A. (2015). Functional representation of vision within the mind: a visual consciousness model based in 3D default space. J. Med. Hypotheses Ideas 9, 45–56. doi: 10.1016/j.jmhi.2015.02.001

[ref56] Jones M. W. Hunt T. (2023). Electromagnetic-field theories of qualia: can they improve upon standard neuroscience? Front. Psychol. 14:1015967. doi: 10.3389/fpsyg.2023.1015967, PMID: 37325753 PMC10267331

[ref57] Kanai R. Tsuchiya N. (2012). Qualia. Curr. Biol. 22, R392–R396. doi: 10.1016/j.cub.2012.03.033 22625852

[ref58] Kanizsa G. (1979). Organization in vision. Essays in gestalt perception. New York: Praeger.

[ref59] Kanizsa G. (1980). Grammatica del vedere. Bologna: Il Mulino.

[ref60] Kanizsa G. (1991). Vedere e pensare. Bologna: Il Mulino.

[ref61] Kent L. Wittmann M. (2021). Time consciousness: the missing link in theories of consciousness. Neurosci. Conscious 2021:niab011. doi: 10.1093/nc/niab011, PMID: 33868714 PMC8042366

[ref62] Kind A. (2008). “How to believe in qualia” in The case for qualia. ed. Wright E. (Cambridge, MA: MIT Press), 285–298.

[ref63] Koch C. (2004). The quest for consciousness: A neurobiological approach. Englewood, CO: Roberts & Company.

[ref64] Koch C. Massimini M. Boly M. Tononi G. (2016). Neural correlates of consciousness: progress and problems. Nat. Rev. Neurosci. 17, 307–321. doi: 10.1038/nrn.2016.22, PMID: 27094080

[ref65] Koenigs M. Young L. Adolphs R. Tranel D. Cushman F. Hauser M.. (2007). Damage to the prefrontal cortex increases utilitarian moral judgements. Nature 446, 908–911. doi: 10.1038/nature05631, PMID: 17377536 PMC2244801

[ref66] Kriegel U. (2004). Consciousness and self-consciousness. Monist 87, 182–205. doi: 10.5840/monist20048725

[ref67] Lamme V. A. (2010). How neuroscience will change our view on consciousness. Cogn. Neurosci. 1, 204–220. doi: 10.1080/17588921003731586, PMID: 24168336

[ref68] Lamme V. A. F. (2020). Visual functions generating conscious seeing. Front. Psychol. 11:83. doi: 10.3389/fpsyg.2020.00083, PMID: 32116908 PMC7034432

[ref69] Lehnertz K. Rings T. Bröhl T. (2021). Time in brain: how biological rhythms impact on EEG signals and on EEG-derived brain networks. Front. Netw. Physiol. 1:755016. doi: 10.3389/fnetp.2021.755016, PMID: 36925573 PMC10013076

[ref70] Lemon R. N. Edgley S. A. (2010). Life without a cerebellum. Brain 133, 652–654. doi: 10.1093/brain/awq030 20305277

[ref71] Levine J. (1983). Materialism and qualia: the explanatory gap. Pac. Philos. Q. 64, 354–361. doi: 10.1111/j.1468-0114.1983.tb00207.x

[ref72] Lewis C. I. (1929). Mind and the world order: Outline of a theory of knowledge, New York: Charles Scribners.

[ref73] Lightman A. Gingerich O. (1992). When do anomalies begin? Science 255, 690–695. doi: 10.1126/science.255.5045.690 17756947

[ref74] Loar B. (2002). “Transparent experience and the availability of qualia” in Consciousness: New philosophical perspectives. eds. Smith Q. Jokic A. (Oxford: Oxford University Press), 173–290.

[ref75] Loorits K. (2014). Structural qualia: a solution to the hard problem of consciousness. Front. Psychol. 5:237. doi: 10.3389/fpsyg.2014.00237, PMID: 24672510 PMC3957492

[ref76] Luccio R. (2011). Gestalt psychology and cognitive psychology. Humana Mente 17, 95–128.

[ref77] Ludwig D. (2023). The functions of consciousness in visual processing. Neurosci. Conscious. 2023:niac018. doi: 10.1093/nc/niac018, PMID: 36628118 PMC9825248

[ref78] Maillé S. Lynn M. (2020). Reconciling current theories of consciousness. J. Neurosci. 40, 1994–1996. doi: 10.1523/JNEUROSCI.2740-19.2020, PMID: 32132221 PMC7055139

[ref79] McFadden J. (2020). Integrating information in the brain’s EM field: the cemi field theory of consciousness. Neurosci. Conscious. 2020:niaa016. doi: 10.1093/nc/niaa016 32995043 PMC7507405

[ref80] McFadden J. (2023). Consciousness: matter or EMF? Front. Hum. Neurosci. 16:1024934. doi: 10.3389/fnhum.2022.1024934, PMID: 36741784 PMC9889563

[ref81] Merleau-Ponty M. (1945). Phénoménologie de la perception. Paris: Gallimard.

[ref82] Metzinger T. (2004). Losing your self being no-one: The self-model theory of subjectivity. Cambridge: The MIT Press.

[ref83] Nagel T. (1974). What is it like to be a bat? Philos. Rev. 83, 435–450. doi: 10.2307/2183914

[ref84] Nani A. Manuello J. Mancuso L. Liloia D. Costa T. Cauda F. (2019). The neural correlates of consciousness and attention: two sister processes of the brain. Front. Neurosci. 13:1169. doi: 10.3389/fnins.2019.01169, PMID: 31749675 PMC6842945

[ref85] Noel J. P. Ishizawa Y. Patel S. R. Eskandar E. N. Wallace M. T. (2019). Leveraging nonhuman primate multisensory neurons and circuits in assessing consciousness theory. J. Neurosci. 39, 7485–7500. doi: 10.1523/JNEUROSCI.0934-19.2019, PMID: 31358654 PMC6750944

[ref86] Northoff G. Klar P. Bein M. Safron A. (2023). As without, so within: how the brain's temporo-spatial alignment to the environment shapes consciousness. Interface Focus 13:20220076. doi: 10.1098/rsfs.2022.0076, PMID: 37065263 PMC10102730

[ref87] Northoff G. Zilio F. (2022). Temporo-spatial theory of consciousness (TTC) - bridging the gap of neuronal activity and phenomenal states. Behav. Brain Res. 424:113788. doi: 10.1016/j.bbr.2022.113788 35149122

[ref88] O’Callaghan C. (2015). The multisensory character of perception. J. Philos. 112, 551–569. doi: 10.5840/jphil20151121035

[ref89] Parvizi J. Damasio A. (2001). Consciousness and the brainstem. Cognition 79, 135–160. doi: 10.1016/S0010-0277(00)00127-X 11164026

[ref90] Polák M. Marvan T. (2018). Neural correlates of consciousness meet the theory of identity. Front. Psychol. 9:1269. doi: 10.3389/fpsyg.2018.01269, PMID: 30087640 PMC6066586

[ref91] Raffone A. Srinivasan N. van Leeuwen C. (2014). Perceptual awareness and its neural basis: bridging experimental and theoretical paradigms. Philos. Trans. R. Soc. B 369:20130203. doi: 10.1098/rstb.2013.0203, PMID: 24639576 PMC3965159

[ref92] Railo H. Koivisto M. Revonsuo A. (2011). Tracking the processes behind conscious perception: a review of event-related potential correlates of visual consciousness. Conscious. Cogn. 20, 972–983. doi: 10.1016/j.concog.2011.03.019, PMID: 21482150

[ref93] Reid T. (1764/1977) in An inquiry into the human mind on the principles of common sense. ed. Brookes D. R. (Edinburgh: Edinburgh University Press)

[ref94] Sanfey J. (2023). Simultaneity of consciousness with physical reality: the key that unlocks the mind-matter problem. Front. Psychol. 14:1173653. doi: 10.3389/fpsyg.2023.1173653, PMID: 37842692 PMC10568466

[ref95] Schwarzkopf D. S. Rees G. (2015). “Perceptual organization and consciousness” in The Oxford handbook of perceptual organization. ed. Wagemans J. (Oxford: Oxford University Press), 799–819.

[ref96] Seager W. (2002). Emotional introspection. Conscious. Cogn. 11, 666–687. doi: 10.1016/s1053-8100(02)00027-2 12470630

[ref97] Searle J. R. (1997). The mistery of consciousness. New York: The New York Review of Books.

[ref98] Searle J. R. (2000). Consciousness. Annu. Rev. Neurosci. 23, 557–578. doi: 10.1146/annurev.neuro.23.1.557 10845075

[ref99] Searle J. R. (2004). Mind. A brief introduction. New York: Oxford University Press.

[ref100] Seth A. K. Bayne T. (2022). Theories of consciousness. Nat. Rev. Neurosci. 23, 439–452. doi: 10.1038/s41583-022-00587-4 35505255

[ref101] Sheinberg D. L. Logothetis N. K. (1997). The role of temporal cortical areas in perceptual organization. Proc. Natl. Acad. Sci. USA 94, 3408–3413. doi: 10.1073/pnas.94.7.3408, PMID: 9096407 PMC20383

[ref102] Shoemaker S. (1991). Qualia and consciousness. Mind C, 507–524. doi: 10.1093/mind/C.400.507

[ref103] Siddharth S. Menon S. (2017). “What does it mean for qualia to be intrinsic?” in Self, culture and consciousness. eds. Menon E. Nagaraj N. Binoy V. (Singapore: Springer)

[ref104] Skokowski P. (2022). Sensing Qualia. Front. Syst. Neurosci. 16:795405. doi: 10.3389/fnsys.2022.795405, PMID: 35359622 PMC8962373

[ref105] Smith D. W. (2018). “Phenomenology” in The Stanford encyclopedia of philosophy (Summer 2018 Edn.). ed. Zalta E. N. Available at: https://plato.stanford.edu/archives/sum2018/entries/phenomenology

[ref106] Solms M. (2021). The hidden spring: A journey to the source of consciousness. New York: Norton.

[ref107] Srinivasan N. (2020). Consciousness without content: a look at evidence and prospects. Front. Psychol. 11:1992. doi: 10.3389/fpsyg.2020.01992, PMID: 32849160 PMC7426455

[ref108] Tegmark M. (2000). Importance of quantum decoherence in brain processes. Phys. Rev. E 61, 4194–4206. doi: 10.1103/PhysRevE.61.4194, PMID: 11088215

[ref109] Todorovic D. (2008). Gestalt principles. Scholarpedia 3:5345. doi: 10.4249/scholarpedia.5345

[ref110] Tomasi C. (2006). “Early vision” in Encyclopedia of Cognitive Science

[ref111] Tononi G. (2008). Consciousness as integrated information: a provisional manifesto. Biol. Bull. 215, 216–242. doi: 10.2307/25470707, PMID: 19098144

[ref112] Tononi G. Edelman G. M. (1998). Consciousness and complexity. Science 282, 1846–1851. doi: 10.1126/science.282.5395.1846 9836628

[ref113] Tononi G. Koch C. (2008). The neural correlates of consciousness: an update. Ann. N. Y. Acad. Sci. 1124, 239–261. doi: 10.1196/annals.1440.004 18400934

[ref114] Tononi G. Koch C. (2015). Consciousness: here, there and everywhere? Philos. Trans. R. Soc. Lond. B Biol. Sci. 370, 1–18. doi: 10.1098/rstb.2014.0167, PMID: 25823865 PMC4387509

[ref115] Tuszynski J. A. (2020). From quantum chemistry to quantum biology: a path toward consciousness. J. Integr. Neurosci. 19, 687–700. doi: 10.31083/j.jin.2020.04.393, PMID: 33378843

[ref116] Tyler C. W. (2020). Ten testable properties of consciousness. Front. Psychol. 11:1144. doi: 10.3389/fpsyg.2020.01144, PMID: 32670141 PMC7326790

[ref117] Van der Werf Y. D. Witter M. P. Groenewegen H. J. (2002). The intralaminar and midline nuclei of the thalamus. Anatomical and functional evidence for participation in processes of arousal and awareness. Brain Res. Brain Res. Rev. 39, 107–140. doi: 10.1016/S0165-0173(02)00181-9, PMID: 12423763

[ref118] Van Gulick R. (2017). “Functionalism and qualia” in The Blackwell companion to Consciousnes. eds. Schneider S. Velmans M. (Oxford, UK: Blackwell), 430–444.

[ref119] Wagemans J. Feldman J. Gepshtein S. Kimchi R. Pomerantz J. R. van der Helm P. A.. (2012). A century of gestalt psychology in visual perception: II. Conceptual and theoretical foundations. Psychol. Bull. 138, 1218–1252. doi: 10.1037/a0029334, PMID: 22845750 PMC3728284

[ref120] Ward L. Guevara R. (2022). Qualia and phenomenal consciousness Arise from the information structure of an electromagnetic field in the brain. Front. Hum. Neurosci. 16:874241. doi: 10.3389/fnhum.2022.874241, PMID: 35860400 PMC9289677

[ref122] Wiese W. (2017). How to solve the problem of phenomenal unity: finding alternatives to the single state conception. Phenom. Cogn. Sci. 16, 811–836. doi: 10.1007/s11097-016-9478-7

[ref123] Williford J. R. von der Heydt R. (2013). Border-ownership coding. Scholarpedia J. 8:30040. doi: 10.4249/scholarpedia.30040, PMID: 25075249 PMC4112134

[ref124] Zeman A. (2001). Consciousness. Brain 124, 1263–1289. doi: 10.1093/brain/124.7.1263, PMID: 11408323

